# Nitrate of Silver

**Published:** 1864-01

**Authors:** John Higginbottom


NITRATE OF SILVER.—*By John Higginbottom*, F. R. S.—I think it very important to call the attention of surgeons to the superiority of ordinary nitrate of silver over the new preparations which have been now some time in use. The new preparation—"lunar caustic points, perfectly tough"—is worthless as an application in surgical cases. It is not nearly so soluble as the old brittle stick of nitrate of silver, and has scarcely any power in checking and subduing inflammation, and usleess in the cure of wounds. The same remarks apply to the cake and crystals of the nitrate of silver used for photographic purposes; which, although they may be more chemimally pure, are much less efficacious for surgical purposes than the old preparations.

It is a remedy to which I called the attention of medical practitioners thirty-seven years since in an "Essay on the use of the nitrate of silver." Every succeeding year it has maintained its value in my estimation; but I fear that if the new preparations continue to be used, it will undeservedly fall into discredit.

The grounds upon which I have formed my opinion are these: I have used the new preparations for some time; and in cases where from past experience I looked forward with a certainty to successful results, I have been much disappointed, and the cause was to me then inexplicable. To give a case: A medical friend had a severe puncture. I applied the nitrate of silver with a conviction that he would have no further trouble with it. To my surprise, the application took little or no effect; surrounding inflammation followed, also of the absorbents. Further applications were made with the same nitrate of silver; but the inflammation continued its usual course, keeping my patient several days in bed, and afterwards it very slowly subsided. Another case: A patient had a severe contused wound on the middle finger of the left hand from a fall. The nitrate of silver was well applied. I expected it would heal under an adherent eschar. That it did not do so surprised and disappointed me. The wound remained some weeks in a painful and inflamed state; and when at last it healed, it left an irritable induration, with swelling. This I treated again and again with the nitrate of silver without much benefit. These and other cases I could relate (one especially, a formidable attack of erysipelas on the leg, which formerly I found yielded to the application, entirely failed), led me to think there must be something wrong in the preparation of the nitrate of silver; and it occurred to me that the new preparation did not produce so much pain as the old one immediately on its application. I procured some of the old-fashioned stick of nitrate of silver. The first application on the case above mentioned did more in removing the irritable, inflamed swelling in four days than all former applications.

From the experience I have had daily of the use of the nitrate of silver for so many years, I am convinced that no remedy of equal power in subduing external inflammation and healing wounds has been discovered, if properly applied, although many remedies have been recommended in lieu of it.

In cases of extensive external inflammation I would use a solution of four scruples of the old-fashioned stick of nitrate of silver to four drachms of distilled water. In common cases of inflammation and wounds, the ordinary stick, as particularly directed in my last work—"Additional observations on the use of nitrate of silver," etc.—*Lancet.*

